# Uncovering the Role of Biological Sex in the Divergent Genetic Profiles of Early and Late-Diagnosed Autism

**DOI:** 10.21203/rs.3.rs-9623041/v1

**Published:** 2026-05-07

**Authors:** Sophie Breunig, Lukas Schaffer, Jeremy Lawrence, Alexander Sheppard, Andrew Grotzinger

**Affiliations:** University of Colorado Boulder; University of Colorado Boulder; University of Colorado Boulder; University of Colorado Boulder; University of Colorado Boulder

**Keywords:** Psychiatric Genetics, Sex-Stratified, Age at Diagnosis, Autism Spectrum Disorder, Genomic SEM

## Abstract

Autism spectrum disorder (ASD) is a neurodevelopmental disorder with a significant male prevalence bias. While recent evidence suggests that genetic heterogeneity is indexed by age at diagnosis, males are also typically diagnosed earlier, such that the extent to which these age-specific findings are confounded by shared genetic signal with sex-specific genetic architecture remains unclear. To test this, we leveraged sex- and age of diagnosis-stratified GWAS summary statistics for ASD and applied a multiple regression framework within Genomic Structural Equation Modeling (Genomic SEM). This approach allowed us to disentangle the overlapping genetic variance between sex, diagnostic timing, and 68 clinically relevant phenotypes reflecting a host of different psychiatric, cognitive, health, and social outcomes.

Consistent with prior findings, we identified significantly divergent genetic associations with external traits between early- and late-diagnosed ASD subtypes. We also identified few findings for sex-specific associations, along with high genetic correlations across these two traits, which questions the biological basis for disproportionate prevalence rates in males. Multiple regression models confirmed that early-specific associations were not confounded by biological sex. Conversely, the correlations between late-diagnosed ASD and the sex-stratified ASD GWAS were near 1, indicating that late-specific associations may be confounded. Follow-up analyses using an internalizing factor provided support for sex differences in clinical presentation and age at diagnosis, with female-diagnosed and late-diagnosed ASD estimated to have stronger genetic correlations with internalizing traits compared to male and early-diagnosed counterparts. This study underscores the importance of subtyping in genetic analyses and provides a framework to disentangle confounded genetic pathways.

## Introduction

Autism spectrum disorder (ASD) is a neurodevelopmental disorder that affects ~ 1% of the global population (Salari et al., 2022) and is diagnosed based on childhood onset of impairment in verbal and nonverbal communication, social skills, as well as the presence of repetitive and restrictive behaviors (American Psychiatric Association, 2013). ASD is one of the most heritable psychiatric disorders, with twin-based heritability estimates of 64% – 91% (Tick et al., 2016) and more recent genome-wide association study (GWAS) estimates of common variant, SNP-based heritability of ~ 12% (Autism Spectrum Disorder Working Group of the Psychiatric Genomics Consortium et al., 2019a). While genetics are clearly critical for understanding the etiology of ASD, it is largely an open question to what extent different stratifying characteristics, such as age at onset and biological sex, may index divergent etiological pathways.

Among individuals with an ASD diagnosis, only approximately 1/5th are girls (Fombonne, 2009; Loomes et al., 2017). Research has shown that boys with ASD present with relatively greater hyperactivity, shorter attention spans, and aggressive behavior (Giarelli et al., 2010; Hartley & Sikora, 2009), while girls present with more communication deficits, sleep problems, and anxious or depressed affect (Hartley & Sikora, 2009). We also note, however, that some of these same studies also report that boys and girls with ASD display a similar pattern of developmental profiles, diagnostic symptoms, and comorbidities (Bitsika et al., 2018; Hartley & Sikora, 2009; Solomon et al., 2012). Differences in prevalence have also been posited to stem from girls’ ability to mask ASD symptoms (American Psychiatric Association, 2013; Tierney et al., 2016), particularly social deficits (Young et al., 2018). In addition to divergent rates of overall diagnosis, girls on the autism spectrum are more likely to be diagnosed later in life than boys (Begeer et al., 2013; Loomes et al., 2017; Rutherford et al., 2016). While age at diagnosis and biological sex have both been posited as outwardly measured stratifying characteristics that may index divergent etiology, relatively delayed diagnoses in girls suggest that age at diagnosis and biological sex are likely confounding each other, making it difficult to determine which of these two stratifying characteristics may be driving identified divergences in etiological pathways.

A recent paper exploring the diverging polygenic and developmental profiles of ASD reported significant etiological heterogeneity in GWAS signal based on the individual’s age of diagnosis (Zhang et al., 2025). The authors conclude that sex differences are unlikely to account for these developmental pathway differences in autism and note that recent other studies finding sex differences in ASD may also partly reflect differences associated with age at diagnosis. For these results to have the greatest impact, it is critical to characterize whether certain ASD risk pathways are, in fact, uniquely indexing age at diagnosis, sex-specific risk, or some combination of both. Beyond the scientific value of this characterization, the potential clinical impact is evidenced by the fact that both stratifying characteristics are associated with disproportionate health burdens. For example, later ASD diagnosis is associated with worse outcomes (Okoye et al., 2023; Vivanti et al., 2014) and hospitalization is more frequent among females with autism (Martini et al., 2022). Parsing the genetic pathways that index ASD diagnosis in females or diagnosed late, particularly with respect to relevant clinical correlates, stands to help guide our understanding of what clinical features may go overlooked for different subpopulations within ASD.

Building on prior work, we aim to move beyond correlations of ASD stratified by age at diagnosis with biological sex to explore more fine-grained associations with relevant comorbidities and external phenotypes, such as psychiatric, interpersonal, cognitive, behavioral, and health traits. We investigate: 1) whether there are notable sex differences in terms of genetic correlations with clinically relevant external phenotypes; 2) if there are divergent genetic patterns between early- and late-diagnosed ASD; and 3) whether these age-at-diagnosis differences are better explained by biological sex. To this end, we included both sex- and age-of-diagnosis-stratified GWAS summary statistics in a multiple regression framework within Genomic SEM to estimate partial associations with clinical correlates. Advantages of examining these stratifying characteristics within a genetic framework include the ability to incorporate mutually exclusive diagnostic characteristics (e.g., early versus late onset; male vs female) within multivariate statistical models to formally disambiguate shared versus unique genetic architecture. The current analyses then systematically test whether genetic relationships between age-stratified ASD and its clinically relevant comorbidities and correlates remain after accounting for genetic variance explained by sex.

## Methods

### Phenotype Selection

#### ASD stratified by sex and age at diagnosis.

ASD GWAS summary statistics stratified by early and late diagnosis, as well as summary statistics stratified by males and females, hereafter referred to as ASD_early_, ASD_late_, ASD_male_, and ASD_female_, were obtained from Zhang et al. (2025). ASD_early_ and ASD_late_ consisted of the multivariate GWAS generated from a Genomic SEM model, representing earlier and later diagnosed ASD across multiple cohorts. The ASD_early_ factor consisted of summary statistics from the Psychiatric Genetics Consortium cohort (The Autism Spectrum Disorders Working Group of The Psychiatric Genomics Consortium, 2017), the SPARK_before6_ cohort (Feliciano et al., 2018), and the iPSYCH_before9_ cohort (Autism Spectrum Disorder Working Group of the Psychiatric Genomics Consortium et al., 2019b). The ASD_late_ factor consisted of summary statistics from SPARK_after10_ (Feliciano et al., 2018), iPSYCH_after10_ (Autism Spectrum Disorder Working Group of the Psychiatric Genomics Consortium et al., 2019b), and FinnGenn (Kurki et al., 2023). More information on the sample characteristics of the individual samples can be found in the original publication that generated the factor GWAS for the ASD_early_ and ASD_late_ phenotypes (Zhang et al., 2025). The effective sample size of ASD_early_ is 33,608 and that of ASD_late_ is 21,775. These sample sizes were calculated by the study authors as the sum of the factor-loading weighted effective sample size for each GWAS included in the factor. ASD_male_ and ASD_female_ consisted of the iPSYCH cohort (Warrier et al., 2022) with 15,025 cases and 19,763 controls for ASD_male_, and 4,845 cases and 19,315 controls for ASD_female_. In the present study, male and female refer to sex assigned at birth.

#### External phenotypes.

Along with the four ASD phenotypes, we included 68 clinically relevant external phenotypes that reflect a host of different psychiatric, cognitive, health, risk-taking, social relationship, and substance use outcomes. For all ASD and external phenotypes, the included GWAS summary statistics of the were of individuals of European-like genetic ancestry and, following the guidelines from the original LDSC developers (Bulik-Sullivan et al., 2015), were sufficiently powered for producing interpretable estimates of genetic correlation, as indicated by a SNP-based heritability *Z*-statistic > 4. A comprehensive list of all included external phenotypes, as well as their corresponding shorthand, is given in **Supplemental Table S1**.

### Quality Control and LD-score Regression

GWAS summary statistics for ASD and all external traits were processed using the “munge” function within the GenomicSEM R package. This standardized quality control procedure restricted the data to HapMap3 SNPs, aligned reference alleles, and applied filters for minor allele frequency (MAF > 1%) and imputation quality (INFO > 0.9). The resulting standardized summary statistics were carried forward to multivariable Linkage Disequilibrium (LD)-score regression (LDSC; Bulik-Sullivan et al., 2015) using LD scores derived from the European-like genetic ancestry subset of the 1000 Genomes Phase 3 project. We exclude the MHC region due to its complex LD structure. Multivariable LDSC yielded a genetic covariance matrix with SNP-based heritabilities on the diagonal and genetic covariances on the off-diagonal, and a sampling covariance matrix with squared standard errors of the estimates and sampling dependencies representing participant sample overlap on the diagonal and off-diagonal respectively. For binary traits, LDSC estimates were converted to a liability scale to account for sample ascertainment and the underlying continuous distribution of genetic risk. This conversion utilized population prevalences from the original GWAS or representative study samples. In addition, we used the sum of effective sample sizes from the contributing cohorts for the ascertainment correction, to produce accurate estimates of the heritability on the liability-scale (Grotzinger et al., 2022). For continuous traits, total sample sizes were used without further correction.

### Genomic SEM

We utilized genomic structural equation modeling (Genomic SEM; Grotzinger et al., 2018) to estimate genetic correlations and multiple regressions for early, late, male and female ASD and the 68 external traits.

#### Constrained vs. unconstrained models.

First, we systematically tested for significant sex differences in the relationship with the external traits by comparing a model where the genetic correlations between ASD_male_ and ASD_female_ were constrained to be equal to a model where they were freely estimated. This produced a model with 1 degree of freedom, such that the χ^2^ statistic for this model specification reflects the level of misfit resulting from the equality constraint. The analyses reflect the model χ^2^ estimates when using the genetic correlation and sampling correlation matrix (i.e., the standardized matrix of sampling dependencies) as input. Thus, significant model χ^2^ estimates represent external traits with significantly different levels of genetic overlap across the ASD_male_ and ASD_female_ subgroups. This same analysis was then repeated for ASD_early_ and ASD_late_ with the same set of external traits. An FDR correction of the standard .05 significance threshold was used to correct for multiple testing across the 68 external traits using the *p.adjust* function in R.

#### Multiple regression models.

Next, we utilized multiple regression analyses to derive partial effects for overlapping ASD subgroups on external traits that exhibited significant sex-based differences. These partial effects indicate whether being diagnosed earlier accounts for the associations of the external trait with being a male or female diagnosed with ASD. Due to strong collinearity between ASD_late_ and both ASD_male_ and ASD_female_ (r_g_male_ = 0.99 [SE = 0.02]; r_g_female_ = 0.91 [SE = 0.02]), the late-diagnosed subgroup was excluded from these models due to limited interpretability, reflecting the low statistical power to disambiguate shared versus unique etiological pathways in the presence of such high genetic correlations. Initial models including ASD_early_, ASD_male_ and ASD_female_, simultaneously as predictors failed to reach significance due to highly overlapping genetic signals. Therefore, we ran separate multiple regression models to test: (1) whether ASD_early_ accounted for the significant relationships between sex-stratified ASD and external traits, and (2) whether ASD_male_ and ASD_female_ separately accounted for the genetic correlations between ASD_early_ and its significantly associated traits. All multiple regression results were FDR corrected for the number of included external traits.

#### Internalizing follow-up analyses.

Many of the external traits that exhibited significantly stronger genetic correlations with ASD_late_ reflect traits in the internalizing domain. Given this trend, we conducted a set of follow-up analyses for an internalizing factor (F_internalizing_). Consistent with the prior literature (Grotzinger et al., 2026), we defined F_internalizing_ using major depressive disorder (MDD; Adams et al., 2025), anxiety disorders (ANX; Purves et al., 2020), and posttraumatic stress disorder (PTSD; Nievergelt et al., 2024). We first confirmed that the greater ASD_late_ overlap held for this latent factor. We then utilized Genomic SEM to determine if shared variance with F_internalizing_ accounted for the divergent associations of ASD_early_ and ASD_late_ with other external traits. Specifically, we specified models where F_internalizing_ predicted both the ASD subtypes and external traits, allowing us to estimate residual associations after removing shared variance with F_internalizing_. Although the significance pattern of stronger genetic overlap with internalizing traits did not replicate in the sex-stratified ASD subtypes, we tested whether leveraging the increased power of the internalizing traits when combined into the internalizing factor would show a significant and larger genetic overlap with one of the sex-stratified ASD subtypes. As with the age at diagnosis stratified ASD subtypes, we then went on to examine whether the significant differences between ASD_male_ and ASD_female_ in their external trait associations were accounted for by the genetic overlap between ASD_female_ and the internalizing space. All analyses were FDR corrected for the number of included external traits.

## Results

### Genetic Correlations are Largely Similar across Sexes

The genetic correlation between ASD_male_ and ASD_female_ was r_g_ = 0.81 (SE = 0.01). Consistent with this high genetic overlap, there were 26 external traits that were significantly correlated with autism in both sexes and were not significantly divergent across sexes. This included similar patterns of genetic overlap with impaired sleep phenotypes, including difficulty waking (r_g_male_ = −0.26 [SE = 0.04]; r_g_female_ = −0.29 [SE = 0.06]; *p*_difference_ = 0.691) and insomnia (r_g_male_ = 0.13 [SE = 0.0]; r_g_female_ = 0.17 [SE = 0.06]; *p*_difference_ = 0.565). Similarly, ASD_male_ and ASD_female_ were both associated with adverse social outcomes, such as reduced ability to confide (r_g_male_ = −0.20 [SE = 0.05]; r_g_female_ = −0.24 [SE = 0.07]; *p*_difference_ = 0.594) and lower family satisfaction (r_g_male_ = −0.37 [SE = 0.06]; r_g_female_ = −0.42 [SE = 0.09]; *p*_difference_ = 0.640).

**Figure 1:**
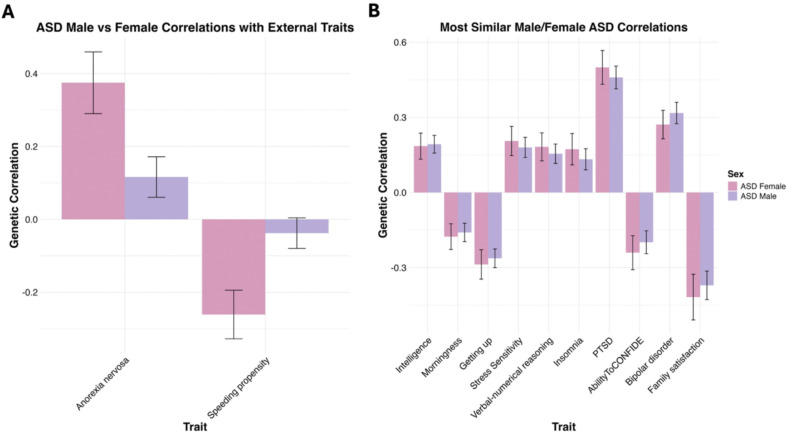
Genetic correlations of ASD_male_ and ASD_female_ with external traits. Panel **A.** displays the significant differences, at an FDR corrected threshold of p <0.05, between ASD_males_ and ASD_female_ with the respective external traits and error bars display the standard error. Panel **B.** displays the top 10 most similar effect estimates of ASD_males_ and ASD_female_ with the external traits and error bars display the standard error. The individual ASD_males_ and ASD_female_ associations with the external traits are significant at an FDR corrected threshold of p <0.05.

While these findings indicate substantial genetic overlap, the correlation between ASD_male_ and ASD_female_ was significantly different from 1, suggesting that sex-stratified ASD subtypes, as currently diagnosed, also capture partially distinct etiological pathways. With that said, significant differences were observed for only two phenotypes. Anorexia nervosa displayed a stronger positive genetic overlap with ASD_female_ (r_g_female_ = 0.38 [SE = 0.09]) compared to ASD_male_ (r_g_male_ = 0.12 [SE = 0.09]; *p*_difference_ = 0.037) and speeding propensity was significantly, negatively associated with only ASD_female_ (r_g_female_ = −0.26 [SE = 0.07]; r_g_male_ = −0.04 [SE = 0.04]; *p*_difference_ = 0.037). These similarities and differences are visualized in Fig. 1, with full results available in **Supplemental Table S2**.

### Late diagnosis shows higher overlap with other psychiatric disorders

**Figure 2:**
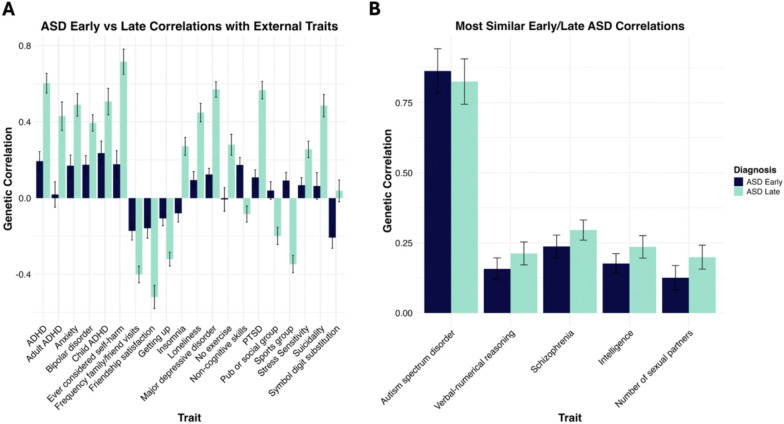
Genetic correlations of ASD_early_ and ASD_late_ with external traits. Panel **A.** displays the 20 most significant differences, at an FDR corrected threshold of p <0.05, between ASD_early_ and ASD_late_ with the respective external traits and error bars display the standard error. Panel **B.** displays the most similar effect estimates of ASD_early_ and ASD_late_ with the external traits and error bars display the standard error. The individual ASD_early_ and ASD_late_ associations with the external traits are significant at an FDR corrected threshold of p <0.05.

The age-at-diagnosis stratified ASD subtypes were more moderately correlated (r_g_ = 0.42; SE = 0.01). This relative genetic independence was underscored by 31 external phenotypes displaying significantly divergent correlations between ASD_early_ and ASD_late_, with a general trend for associations to be stronger with ASD_late_ (Fig. 2; **Supplementary Table S3**). This included much higher degrees of genetic overlap with a range of psychiatric disorders, including MDD (r_g_early_ = 0.12 [SE = 0.03]; r_g_late_ = 0.57 [SE = 0.04]; *p*_difference_ = 1.60E-19), PTSD (r_g_early_ = 0.11 [SE = 0.04]; r_g_late_ = 0.57 [SE = 0.05]; *p*_difference_ = 4.72E-15), and ADHD (r_g_early_ = 0.19 [SE = 0.05]; r_g_late_ = 0.60 [SE = 0.05]; *p*_difference_ = 9.10E-15). Notably, the larger genetic overlap for ASD_late_ with ADHD was observed for ADHD diagnosed in both adulthood (r_g_early_ = 0.02 [SE = 0.07]; r_g_late_ = 0.43 [SE = 0.07]; *p*_difference_ = 2.06E-6) and childhood (r_g_early_ = 0.24 [SE = 0.06]; r_g_late_ = 0.51 [SE = 0.07]; *p*_difference_ = 3.22E-4). ASD_late_ was also more strongly linked to adverse social outcomes, including significantly higher levels of loneliness (r_g_early_ = 0.09 [SE = 0.04]; r_g_late_ = 0.45 [SE = 0.05]; *p*_difference_ = 2.47E-9), and negative associations with friendship (r_g_early_ = −0.16 [SE = 0.05]; r_g_late_ = −0.52 [SE = 0.06]; *p*_difference_ = 3.72E-6) and family relationship satisfaction (r_g_early_ = −0.23 [SE = 0.05]; r_g_late_ = −0.43 [SE = 0.06]; *p*_difference_ = 9.02E-3).

A distinct pattern also emerged regarding cognitive and educational profiles: ASD_early_ was more positively associated with educational attainment (r_g_early_ = 0.23 [SE = 0.03]; r_g_late_ = 0.10 [SE = 0.03]; *p*_difference_ = 7.67E-4) and its non-cognitive components (r_g_early_ = 0.17 [SE = 0.04]; r_g_late_ = −0.08 [SE = 0.04]; *p*_difference_ = 6.20E-7), whereas ASD_early_ was negatively associated with memory (r_g_early_ = −0.09 [SE = 0.05]; r_g_late_ = 0.08 [SE = 0.05]; *p*_difference_ = 5.65E-03) and symbol digit substitution (r_g_early_ = −0.21 [SE = 0.06]; r_g_late_ = 0.04 [SE = 0.06]; *p*_difference_ = 6.28E-4) respectively. These findings suggest that while the early-diagnosed subtype is more closely tied to cognition and school-performance outcomes, the late-diagnosed subtype is characterized by increased psychiatric burden.

A much smaller subset of five traits was comparably associated with both subgroups, including general ASD diagnosis (r_g_early_ = 0.86 [SE = 0.08]; r_g_late_ = 0.83 [SE = 0.08]; *p*_difference_ = 0.62). Additionally, both subtypes shared significant genetic overlap with measures of general intelligence (r_g_early_ = 0.18 [SE = 0.04]; r_g_late_ = 0.24 [SE = 0.04]; *p*_difference_ = 0.21) and verbal numerical reasoning (r_g_early_ = 0.16 [SE = 0.08]; r_g_late_ = 0.21 [SE = 0.04]; *p*_difference_ = 0.28).

### Female-specific associations cannot be explained by early diagnosis

External traits showing significantly divergent correlations with ASD_male_ and ASD_female_ were further analyzed in multiple regressions with ASD_early_. Due to its near-perfect genetic overlap with ASD_male_ and substantial genetic overlap with ASD_female_, ASD_late_ was excluded from the multiple regression analyses. Both divergent traits (anorexia nervosa and speeding propensity) were significantly associated with ASD_female_, and only anorexia nervosa was associated with ASD_male_. In multiple regression models with ASD_male_ and ASD_early_, the univariately significant (although smaller than the female association) positive male association with anorexia nervosa (*p*_male_ = 3.69E-02) was attenuated to non-significance (*p*_male_ = 8.21E-01).

In multiple regression models with ASD_female_ and ASD_early_ as correlated predictors, only ASD_female_ was positively associated with anorexia nervosa (*p*_female_ = 2.21E-3) and negatively associated with speeding propensity (*p*_female_ = 2.16E-3). These results, provided in **Supplemental Tables S4 and S5**, suggest that these specific associations are driven by female-stratified ASD liability, rather than the timing of diagnosis.

### Early-specific associations cannot be explained by male or female ASD

While there were far more late-specific associations when compared to early diagnosis, the correlations between ASD_late_ and ASD_male_ and ASD_female_ were so high (> .9) that follow-up multiple regressions models were underpowered. This observation is, in and of itself, important to keep in mind when interpreting late-specific results above, wherein current GWAS do not allow for definitively ruling out confounding by sex. Among the effects that were more significantly associated with ASD_early_, non-cognitive aspects of educational attainment and the symbol-digit task remained significant and increased in magnitude when controlling for male or female signal, indicating that these traits may be specific clinical hallmarks of the early-diagnosis phenotype. Among the effects that were more significantly associated with ASD_late_, but still showed significant relationships with ASD_early_, including either ASD_male_ or ASD_female_ often made the ASD_early_ relationships nonsignificant. This indicates that many of the significant correlations with ASD_early_ reflect overlapping pathways that are nonspecific to this subgroup within ASD (**Online Supplement**). Conversely, other ASD_early_ associations consistently became strengthened when including either sex-stratified ASD, indicating that signal unique to ASD_early_ might be partially masked by genetic overlap with more general ASD processes shared across males or females.

### Internalizing follow-up analyses

**Figure 3:**
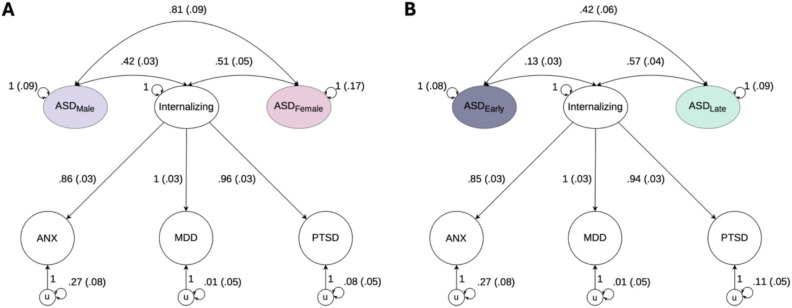
Path diagrams of F_internalizing_ and its unconstrained genetic correlations with the ASD subtypes. Panel **A.** displays F_internalizing_ and its unconstrained genetic correlations with ASD_male_ and ASD_female_ as modeled in genomic structural equation modeling. Panel **B.** displays F_internalizing_ and its unconstrained genetic correlations with ASD_early_ and ASD_late_ as modeled in genomic structural equation modeling. In the model, F_internalizing_ is a common genetic factor of the genetic components of ANX, MDD, and PTSD and u is the residual genetic variance in these phenotypes that is not explained by the internalizing factor. Observed variables are represented as squares, and latent variables are represented as circles. The genetic component of each phenotype is represented with a circle because the genetic component is a latent variable that is not directly measured but is inferred using linkage disequilibrium score regression. Single-headed arrows are regression relations; double-headed arrows connecting back to the same origin are variances; and double-headed arrows connecting 2 variables are correlations. ANX, Anxiety; MDD, Major depressive disorder; PTSD, Post-traumatic stress disorder.

Divergent genetic correlations for age-stratified ASD were primarily concentrated in internalizing traits, which exhibited significantly stronger associations with ASD_late_ than ASD_early_. This pattern persisted in multiple regressions, where sex-stratified signals significantly attenuated the relationship between early-diagnosed ASD and the internalizing space. These phenotypes, including MDD, PTSD, anxiety, measures of self-harm, and measures of social satisfaction, accounted for nearly half of the divergent traits identified across diagnostic ages. Follow-up models using a latent internalizing factor (F_internalizing_) confirmed these trends. The χ^2^ difference test revealed that F_internalizing_ was significantly more correlated with ASD_late_ then ASD_early_ (r_g_late_ = 0.57 [0.04] and r_g_early_ = 0.13 [0.03], p_difference_ = 1.33E-27; Fig. 3). Furthermore, while individual internalizing disorders did not exhibit significant sex differences, leveraging the increased power of their shared signal in the latent factor revealed a stronger association with ASD_female_ (r_g_female_ = 0.51 [0.06] and r_g_male_ = 0.42 [0.03], p_difference_ = 4.62E-2). However, as this χ^2^ difference test was only marginally significant, this result should be interpreted with caution.

#### Increased number of sex-stratified differences after accounting for Internalizing.

Since the internalizing factor showed divergent genetic associations across the sex-stratified ASD phenotypes, we further tested whether significant differences in associations with external traits are accounted for by their genetic overlap with F_internalizing_. After adjusting for F_internalizing_, several external traits showed significant sex-stratified differences. Speeding propensity and anorexia nervosa, which were significantly differently associated with ASD_male_ and ASD_female_ in unadjusted analyses, remained significantly different when accounting for internalizing liability (p_difference_ < 0.05). Specifically, for speeding propensity, the female correlation was attenuated (*β*_unadjusted_ = −0.26 [SE = 0.07] → *β*_adjusted_= −0.14 [SE = 0.07]), while the male correlation shifted from negative to positive (*β*_unadjusted_ = −0.03 [SE = 0.04] → *β*_adjusted_= 0.07 [SE = 0.04]). For anorexia nervosa the association decreased for females (*β*_unadjusted_ = 0.38 [SE = 0.08] → *β*_adjusted_= 0.22 [SE = 0.08]) and was eliminated completely for males (*β*_unadjusted_ = 0.12 [SE = 0.06] → *β*_adjusted_= −0.01 [SE = 0.06]). Notably, accounting for F_internalizing_, unmasked significant sex differences for ADHD and number of sexual partners, which were non-significantly different in univariate models. While the association with ADHD was reduced for both sexes, the attenuation was more pronounced in females (*β*_unadjusted_ = 0.39 [SE = 0.07] → *β*_adjusted_ = 0.07 [SE = 0.07]) than in males (*β*_unadjusted_ = 0.51 [SE = 0.05] → *β*_adjusted_ = 0.23 [SE = 0.05]), resulting in a significant divergent association (p_difference_ < 0.05).

#### Reduced number of diagnostic age stratified differences after accounting for Internalizing.

**Figure 4:**
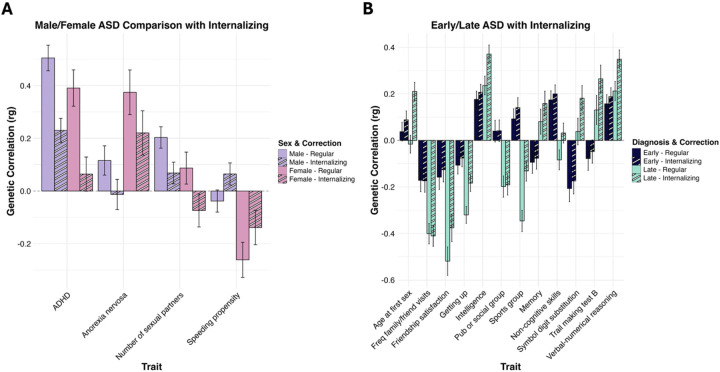
Comparison of ASD subtype genetic correlations with external traits with and without accounting for genetic overlap with F_internalizing_. Panel **A.** displays ASD_male_ and ASD_female_ genetic correlations with external traits before and after accounting for F_internalizing_. Panel **B.** displays ASD_early_ and ASD_late_ genetic correlations with external traits before and after accounting for F_internalizing_. Results without accounting for the overlap of the internalizing factor with ADHD are displayed in the solid bars, and results accounting for the overlap of the internalizing factor with ADHD are displayed in the striped bars. Error bars display the standard error.

Similar to the sex-stratified analyses, we tested whether genetic overlap with F_internalizing_ accounted for the divergent trait associations between ASD_early_ and ASD_late_. After controlling for internalizing liability, the number of significantly different traits was reduced from 31 to 12. Nine of these traits, including social factors (frequency of family visits, friendship satisfaction, and loneliness traits), cognitive tasks (symbol digit, memory, TMTB), and non-cognitive aspects of educational attainment remained significantly different between the two subtypes (p_difference_ < 0.05). Conversely, 19 previously divergent associations were attenuated to non-significance. Most notably, childhood and adulthood ADHD no longer showed significant differences between diagnostic ages after accounting for F_internalizing_. For childhood ADHD, correlations were reduced for both subtypes, but more substantially for ASD_late_ (*β*_unadjusted_ = 0.51 [SE = 0.07] → *β*_adjusted_= 0.23 [SE = 0.07]) than ASD_early_ (*β*_unadjusted_ = 0.24 [SE = 0.06] → *β*_adjusted_= 0.17 [SE = 0.06]). A similar trend occurred for adulthood ADHD, where ASD_late_ associations were nearly eliminated (*β*_unadjusted_ = 0.42 [SE = 0.07] → *β*_adjusted_= 0.02 [SE = 0.07]). Interestingly, new significant differences emerged for age at first sex, intelligence, and verbal-numerical reasoning. These traits showed a pattern of increased genetic correlations for both subtypes, with the increase being more pronounced in ASD_late_. Full results of genetic correlations of age at diagnosis and sex-stratified ASD with external traits adjusted for F_internalizing_ are provided in **Supplemental Tables S8 and S9** and visualized in Fig. 4.

## Discussion

The present study utilized Genomic SEM to disentangle the intertwined genetic effects underlying biological sex and age at diagnosis in autism spectrum disorder. We demonstrate that, although these groups represent subtypes of the same disorder, they possess overlapping and distinct genetic qualities. Notably, the genetic correlation between ASD_male_ and ASD_female_ (r_g_ = 0.81) was substantially stronger than that between ASD_early_ and ASD_late_ (r_g_ = 0.42), which is mirrored in their associations with external traits. While sex-stratified subtypes showed minimal divergence, the early and late-diagnosed subtypes differed significantly across nearly half of the included social, cognitive, and psychiatric domains. Because many of these traits can be described as correlates or indicators of the internalizing dimension, we then went on to examine genetic overlap of the ASD subtypes with a latent internalizing factor. Analyses revealed larger, positive relationships for internalizing with ASD_female_ and ASD_late_. Accounting for this internalizing overlap attenuated several previously observed subgroup differences, notably reducing the genetic association between diagnostic age in ASD and adulthood ADHD to near zero. Interpretations of these findings are discussed in detail below.

### Genetic Correlations with External Traits

In examining sex-stratified genetic correlations, anorexia nervosa and speeding propensity emerged as the only significantly divergent individual traits. While anorexia was more strongly positively associated with ASD_female_, speeding propensity was more strongly negatively associated with ASD_female_. In line with phenotypic research indicating higher internalizing symptoms in women (Duvekot et al., 2017; Hartley & Sikora, 2009), we also find that an internalizing factor is slightly more associated with ASD_female_. The fact that we did not identify this trend using individual internalizing disorders points to the value of leveraging shared signal through multivariate modeling.

The age-at-diagnosis subtypes displayed a much lower genetic correlation than those stratified by sex. In fact, the genetic overlap between ASD_early_ and ASD_late_ was lower than between ASD_late_ and several other psychiatric disorders including MDD, ADHD, and PTSD. The ASD_early_ and ASD_late_ correlation is also about half the size of the genetic overlap between ADHD diagnosed early and late (Breunig et al., 2024), suggesting particular divergence between the early and late ASD phenotype relative to other neurodevelopmental conditions. This low correlation was reflected in 31 traits that significantly differentiated the two groups, with later diagnosis consistently associated with greater psychiatric burden, particularly in the internalizing space.

This mirrors the findings by Zhang et al. (2025), who found the same pattern of later diagnosed ASD with greater mental health problems. They additionally find no significant differences between early and late diagnosis with a host of developmental milestones and phenotypes, except for delays in expressive vocabulary and age at walking, which were both more strongly associated with early diagnosed ASD. The relative lack of divergence in early developmental milestones suggests that late diagnosis is not simply a matter of “milder” childhood symptoms that only become salient once the environmental and occupational demand is high enough (Fusar-Poli et al., 2017). In addition, other factors may result in an increased genetic overlap between ASD_late_ and psychiatric conditions. First, diagnostic misclassification may occur if other conditions are incorrectly identified as autism during adulthood due to the challenges of retroactive symptom assessment (Fusar-Poli et al., 2022; Zhang et al., 2025). Second, if both disorders (autism diagnosis and a co-occurring condition) are present, this can lead to diagnostic overshadowing, where the presence of co-occurring mental-health conditions can delay an autism diagnosis due to the clinical complexity of the interacting conditions (Fusar-Poli et al., 2022; Zhang et al., 2025).

The early vs. late ASD findings also produce markedly similar patterns of results to early vs. late diagnosed ADHD (Breunig et al., 2024), where adulthood presentations of both disorders show stronger genetic correlations with major depression, suicidality, and loneliness. Based on previous ADHD research, we hypothesized that age-of-diagnosis differences might be partially confounded by sex, specifically, that stereotypically male symptom profiles facilitate earlier diagnosis, while female-typical presentations often lead to later identification (Breunig et al., 2024). The sex and age of diagnosis stratified ASD data combined with the multiple regression modeling capabilities of Genomic SEM allowed us to test this hypothesis in the context of ASD. We find that the few early-specific associations were not confounded by biological sex. Conversely, ASD_late_ was too highly correlated with the sex-stratified subgroups to permit estimating the multiple regression models in this dataset. We also find that the two female-specific associations were not confounded by early diagnosis, although the effects of a stronger associated late diagnosis remains to be determined once we have more power to detect this. This necessitates better powered studies and, in the meantime, interpreting with caution claims that late diagnosis uniquely indexes greater genetic overlap with external traits, such as other psychiatric disorders.

### Internalizing Follow-Up Models Reveal Possible Mechanisms for Divergence

The latent internalizing factor, F_internalizing_, showed stronger genetic associations with ASD_female_ and ASD_late_ than their respective counterparts. These elevated associations likely reflect higher rates of psychiatric comorbidity in these populations Martini et al., 2022), potentially driven by the increased liability for secondary internalizing problems when individuals navigate school and social systems without early diagnosis or accommodation (Clark et al., 2018). While diagnostic misclassification in adulthood remains a possibility, the emergent etiological picture suggests that later-diagnosed and female-stratified ASD are uniquely characterized by higher genetic overlap with internalizing phenotypes.

Accounting for internalizing liability within sex-stratified models clarified several distinct patterns of association. While significant sex differences for anorexia nervosa and speeding propensity persisted, controlling for F_internalizing_ unmasked a previously unobserved difference in ADHD associations. ADHD, which was previously non-significantly different in its association with ASD_male_ and ASD_female_, became significantly different when accounting for F_internalizing_. Specifically, the genetic link between ASD and ADHD in females was significantly more explained by internalizing liability than in males. This indicates that the ASD-ADHD relationship in women is more closely tied to general psychiatric distress than it is in men, providing a more nuanced understanding of female-specific comorbidities. Similarly, internalizing liability accounted for much of the divergent signal seen in age-at-diagnosis models. F_internalizing_ explained the larger genetic overlap between ASD_late_ and clinical correlates of internalizing, such as loneliness and suicidal behavior. This mirrors prior findings for age-at-diagnosis stratified ADHD, which showed a stronger attenuation of both late diagnosed ADHD with loneliness and suicidality (Breunig et al., 2024). Notably, the genetic association of both ASD_early_ and ASD_late_ with adulthood ADHD disappeared entirely after controlling for F_internalizing_, suggesting that this genetic link is fully accounted for by overlap with psychiatric distress, rather than ASD-specific pathways. This pattern was consistent across several psychiatric traits, which were nonsignificant once general internalizing liability was accounted for. This may reflect the fact internalizing captures particularly transdiagnostic general tendencies (e.g., distress tolerance) that then broadly genetically mediates the link between ASD and other disorders.

Conversely, the genetic associations between ASD subtypes and cognitive tasks increased in magnitude after accounting for shared genetic signal with internalizing disorders. This “unmasking” of cognitive signal suggests a clear etiological divergence between ASD and internalizing disorders regarding cognitive tasks. These findings support cognitive performance as a key stratifying characteristic for making a differential diagnosis between ASD and internalizing disorders that can share clinical characteristics (e.g., reduced social motivation).

### Limitations, Future Directions, and a Note on Subtyping

While this framework provides a tool for disentangling subtype specific signal in ASD, it is not without limitations. The high genetic correlation between ASD_late_ and sex-stratified GWAS summary statistics limited our ability to fit a comprehensive four-predictor model. This would have more definitively isolated the interactions between diagnostic age and biological sex. Larger sample sizes for the subgroups are necessary to increase power for detecting subtle differences between sex and the late diagnosed group. In addition, stratifying external traits by diagnosis and sex may identify unique links that are otherwise masked by aggregated GWAS that, for example, include biological sex as a covariate. Future research should prioritize larger sample sizes, sex-stratified external trait, and ancestrally diverse cohorts to increase statistical power and generalizability.

This study not only sheds light on the intertwined mechanisms leading to an autism diagnosis, or a lack thereof, but also highlights the importance of subtyping in psychiatric genetics. Lumping heterogeneous symptom profiles into a single diagnostic category risks masking key genetic information. To gain further insight into disease etiology, we need to identify underlying symptoms that are shared among similar disorders, and can distinguish between different disorders and their subtypes (Derks et al., 2022). While sample sizes are often insufficient for symptom-level analyses, stratifying by age of onset or diagnosis (Rajagopal et al., 2022), biological sex (Hu et al., 2025; Silveira et al., 2023), and codified clinical subtype (e.g., Bipolar-I vs II; Lawrence et al., 2024) serves as a necessary middle ground. The high but imperfect genetic correlations between these subtypes along with downstream analyses of their differences highlight nuances between the subtypes that, if leveraged correctly, could lead to more accurate diagnostic processes.

### Conclusion

This study demonstrates potential source of genetic heterogeneity within ASD, with age at diagnosis serving as a more distinct genetic stratifier than biological sex. Our findings highlight that individuals diagnosed later in life carry a disproportionate genetic burden for other psychiatric disorders, a profile that is partially distinct from the early-diagnosed ASD presentation. At the same time, current findings indicate that this cannot be conclusively disambiguated from confounded sex-stratified signal in ASD. Identifying the source of subgroup differences remains challenging, as age at diagnosis and sex are not independent of one another; rather, they are highly correlated and frequently confounded by sex-specific presentations that are traditionally regarded as hallmarks of developmental disorders like ASD. Moving forward, future versions of these results could be used to develop more informed diagnostic approaches that consider the correlates and features of individuals that are currently being diagnosed late or females with ASD that are likely being underdiagnosed.

## Supplementary Material

Supplementary Files

This is a list of supplementary files associated with this preprint. Click to download.

• v1OnlineSupplement.docx

• v3SupplementalTables.xlsx

## Data Availability

GWAS summary statistics for all stratified ASD traits are cited in the main text. Age at diagnosis stratified ASD summary statistics were obtained here: https://figshare.com/articles/dataset/Summary_statistics_for_Polygenic_and_developmental_profiles_of_autism_differ_by_age_at_diagnosis/29566052/2. Sex stratified ASD summary statistics were obtained from the authors of the manuscript, as specified here: https://www.nature.com/articles/s41586-025-09542-6. GWAS summary statistics for all included external traits are cited in the Supplementary Table S1.
